# Distinguishing Between Healthy and Unhealthy Newborns Based on Acoustic Features and Deep Learning Neural Networks Tuned by Bayesian Optimization and Random Search Algorithm

**DOI:** 10.3390/e27111109

**Published:** 2025-10-27

**Authors:** Salim Lahmiri, Chakib Tadj, Christian Gargour

**Affiliations:** 1 Department of Supply Chain and Business Technology Management, John Molson School of Business, Concordia University, Montreal, QC H3G 1M8, Canada; 2 Department of Electrical Engineering, École de Technologie Supérieure, Montreal, QC H3C 1K3, Canada; chakib.tadj@etsmtl.ca (C.T.); christian.gargour@etsmtl.ca (C.G.)

**Keywords:** newborn cry, Mel-frequency cepstral coefficients, auditory-inspired amplitude modulation, prosody, deep feedforward neural networks, Bayesian optimization, random search optimization

## Abstract

Voice analysis and classification for biomedical diagnosis purpose is receiving a growing attention to assist physicians in the decision-making process in clinical milieu. In this study, we develop and test deep feedforward neural networks (DFFNN) to distinguish between healthy and unhealthy newborns. The DFFNN are trained with acoustic features measured from newborn cries, including auditory-inspired amplitude modulation (AAM), Mel Frequency Cepstral Coefficients (MFCC), and prosody. The configuration of the DFFNN is optimized by using Bayesian optimization (BO) and random search (RS) algorithm. Under both optimization techniques, the experimental results show that the DFFNN yielded to the highest classification rate when trained with all acoustic features. Specifically, the DFFNN-BO and DFFNN-RS achieved 87.80% ± 0.23 and 86.12% ± 0.33 accuracy, respectively, under ten-fold cross-validation protocol. Both DFFNN-BO and DFFNN-RS outperformed existing approaches tested on the same database.

## 1. Introduction

Voice is essential to recognize some characteristics, including gender, illness, mental distress, and physical pain. Nowadays, the analysis of pathological voice is becoming important in healthcare for the diagnosis of pathologies [1,2]. Indeed, computer-aided diagnosis (CAD) systems were proposed to detect various pathologies by analysis and classification of voice records, for instance, to detect autism [3], dysphagia [4], brain lesion [5], COVID-19 [6,7], menarcheal status [8], dysarthria [9], laryngeal disorder [10,11], multiple sclerosis [12], dysphonia [13], spasmodic dysphonia and recurrent laryngeal nerve palsy [14], and Parkinson’s disease [15,16,17,18].

In this regard, voice change is also considered a clinical symptom of early detection of an unhealthy newborn, and it is easy to record by using a microphone and a recorder. Indeed, it is a non-invasive monitoring approach for the diagnosis of newborns. Therefore, several CADs were proposed in the literature to automatically analyze and classify cry records to distinguish between healthy and unhealthy newborns. For example, Rosales–Pérez et al. [19] used Mel frequency cepstral coefficients and Linear predictive coding for feature extraction and genetic selection of a fuzzy model for classification of normal and pathological cry. The proposed automatic system obtained 99.42% accuracy. Chittora et al. [20] used spectrogram-based energy features to examine ten different types of infant cry pathologies. The support vector machine (SVM) classifier achieved 98.22% accuracy. Sachin et al. [21] designed an automatic system to detect neonatal asphyxia based on support vector machines trained with Mel frequency cepstrum. The proposed model yielded 92% accuracy. Lim et al. [22] extracted energy features from the dual-tree complex wavelet packet transform representation of the infant cry signal and employed different methods of feature selection to identify energy features to be fed to the extreme learning machines. They obtained 97.87% accuracy in the classification of asphyxia versus normal, 100% accuracy in the classification of deaf versus normal, and 87.26% accuracy in the classification of hunger versus pain. Anders et al. [23] studied the performance of the convolution neural networks in the classification of infant vocalization sequences, including crying, fussing, babbling, laughing, and vegetative vocalizations. The convolutional neural networks yielded 72% accuracy when trained with spectrograms. Ashwini et al. [24] proposed a system to classify neonatal cries into pain, hunger, and sleepiness. First, the short-time Fourier transform was applied to the neonatal cry record to obtain its spectrogram image. Second, the spectrogram is processed by a deep convolutional neural network to extract its deep features. Finally, the support vector machine was employed for classification. Trained with a radial basis function, the support vector machine achieved 88.89% accuracy. Ting et al. [25] investigated the use of hybrid features of MFCC, chromagram, Mel-scaled spectrogram, spectral contrast, and Tonnetz and deep learning models in classifying asphyxia cry in infant subjects. They found that convolution neural networks performed better than deep neural networks when all were trained with MFCC features. In addition, the deep neural network models performed better with hybrid features compared to those with a single feature of MFCC. Furthermore, the deep neural network model with multiple hidden layers achieved an accuracy of 100% in classifying normal and asphyxia cry, and 99.96% for non-asphyxia and asphyxia cry when the hybrid features were used. Abbaskhah et al. [26] trained the support vector machine, multilayer perceptron, and convolutional neural Network classifiers with Mel frequency cepstral coefficients and entropy to distinguish between five classes of infant cries. Without the synthetic minority over-sampling technique, the support vector machine, multilayer perceptron, and convolutional neural Network classifiers, respectively, achieved 82.3%, 87.6%, and 92.1% average accuracy. With the synthetic minority over-sampling technique, they, respectively, obtained 86.1%, 89.2%, and 91.1% average accuracy. In an interesting study, Ozseven [27] investigated the effect of using hand-crafted features and spectral images individually and in a hybrid in the classification of infant cries. The results show that texture analysis methods are insufficient and that hand-crafted feature sets and spectrogram and scalogram images provide high success. The pre-trained deep learning models ShuffleNet and ResNet-18 achieved, respectively, 97.57% and 95.17% accuracy by using scalogram features. Lahmiri et al. [28] proposed a deep feedforward neural network (DFFNN) trained with cepstrum analysis-based coefficients. It outperformed both the SVM and naïve Bayes when applied to expiration and inspiration infant cry signals. Specifically, it achieved very close to perfect accuracy and perfect accuracy when tested on expiration and inspiration sets. In addition, Lahmiri et al. [29] trained DFFNN, long short-term memory (LSTM) neural networks, and convolutional neural networks (CNN) with cepstrum analysis-based coefficients. All validated on expiration and inspiration sets separately, it was found that CNN achieved the highest accuracy, followed by DFFNN.

Recently, acoustic features including Mel frequency cepstral coefficients (MFCC), auditory-inspired amplitude modulation (AAM) features, and a prosody feature set of tilt, intensity, and rhythm features were employed to train machine learning models to distinguish between healthy and unhealthy cries of newborns. For instance, Matikolaie et al. [30] found that the SVM and probabilistic neural networks (PNN) achieved the best accuracy when trained by both AAM and MFCC features. They, respectively, obtained 78.70% and 72.8% accuracy. Lahmiri et al. [31] applied the chi-square test to each set of features to retain the ten most significant ones used as biomarkers and used the Bayesian optimization (BO) method to optimize the hyperparameters of the support vector machine (SVM) with radial basis function (RBF) kernel and k-nearest neighbors (kNN). They found that the SVM trained with AAM features achieved 83.62% accuracy, and the kNN trained with MFCC, AAM, and prosody obtained 82.88%.

**Figure 1:**
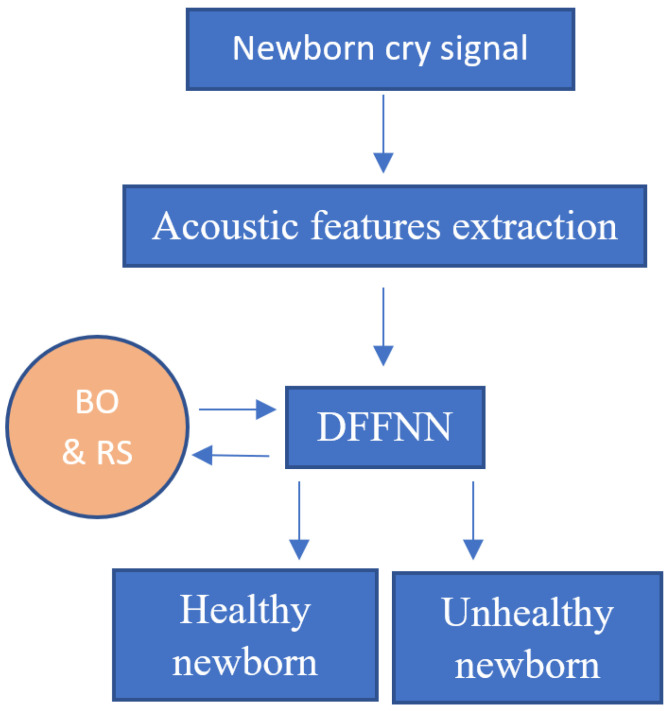
Flowchart of the CAD system for newborn classification based on deep learning (for instance, deep feedforward neural network/DFFNN) trained with acoustic features (MFCC, AAM, and prosody). The configuration of the DFFNN is tuned by using Bayesian optimization (BO) and random search (RS) algorithms.

The main purpose of our study is to improve the classification accuracy by using deep feedforward neural networks (DFFNN) [32] and compare the results with those obtained in [30,31]. Specifically, using the same database as in [30,31], we implement DFFNN and optimize its configuration by using two different optimization algorithms, namely, the Bayesian optimization (BO) [33,34] and random search (RS) [35,36]. Indeed, fine-tuning parameters is a time-consuming task and is needed in machine learning since it affects the accuracy of the models. Therefore, each DFFNN-BO and DFFNN-RS system is trained by using separately each type of acoustic features (MFCC, AAM, and prosody) or all of them. The DFFNN is chosen as it was found to be effective in previous works related to the classification of newborn cry signals [28,29]. Indeed, the DFFNN trained with acoustic features is expected to achieve higher accuracy thanks to the powerful ability of deep learning to learn data. In addition, by training the DFFNN with different sets of acoustics, one could determine what kind of features help obtain the highest accuracy and explain the results. Although BO and RS are selected for hyperparameter tuning as they are effective for global optimization of black-box functions and do not require a long processing time. Figure 1 shows the flowchart of the proposed newborn cry signal analysis and classification.

The key contributions of this paper are as follows:

(1) The DFFNN was found to be effective when trained with cepstrum-based coefficients in previous works [28,29]. This study investigates the gaps in existing DFFNN-based newborn cry record classification.
(2) The experiments are conducted by highlighting the importance of acoustic features on the performance of the DFFNN and addressing the explanation of its performance.
(3) We investigate the contribution of two optimization methods (Bayesian optimization versus random search) in improving the accuracy of DFFNN.
(4) By comparing the performance of the DFFNN-BO and DFFNN-RS systems, both outperformed models in [30,31] on the same database, proving that they can be used in newborn cry signal classification with an increase in explanatory power.

The content of this article is structured as follows. Section 2 introduces the methods. In Section 3, the dataset and experiment settings are introduced, and the experimental results are presented. Section 4 discusses the results. Finally, Section 5 concludes the paper.

## 2. Methods

### 2.1. Acoustic Features

#### 2.1.1. Mel Frequency Cepstral Coefficients (MFCC)

The MFCC are short-term acoustic features suitable for finding the critical bandwidth used by the human auditory system to recognize different tones based on the Mel scale, which is given by:

(1) $$M\left(f\right)=1125\times log\left(1+\frac{f}{700}\right)$$

where f and M(f) denote the frequency value of the signal and its corresponding Mel value. The MFCC are obtained following five steps [37]: (1) framing (voice signal is broken down into overlapping frames), (2) windowing (each frame is multiplied by a Hamming window), (3) applying Fast Fourier transform (convert the signal to the frequency domain and calculate its periodogram), (4) applying Mel filter banks (compute the average of each spectral power density contained in each filter and computes its logarithm), and (5) converting from cepstral to temporal domain by calculating the inverse discrete Fourier transform.

#### 2.1.2. AAM Features

The extraction of AAM features is based on five consecutive steps [38]. First, the newborn cry signal is processed by a short-time discrete Fourier transform (STDFT). Second, the squared magnitudes of the resulting acoustic frequency components are categorized into 27 sub-bands for better characterization of the original cry signal. Third, a second transform is performed across time for each of the 27 sub-band magnitude signals. Fourth, a band-pass filter is applied to the grouped squared modulation frequencies. Finally, the logarithm transformation is applied for compression purposes.

#### 2.1.3. Prosody Features

The prosody set is composed of the tilt feature subset, the intensity feature subset, and the rhythm feature subset. Tilt features are estimated based on two parameters: A_t_ and D_t_, they are defined as follows:

(2) $$A_{t}=\left(\frac{\left|A_{r}\right|-\left|A_{f}\right|}{\left|A_{r}\right|+\left|A_{f}\right|}\right)$$

(3) $$D_{t}=\left(\frac{\left|D_{r}\right|-\left|D_{f}\right|}{\left|D_{r}\right|+\left|D_{f}\right|}\right)$$

where *A_f_* is the amplitude of the contours of the fundamental frequency *F*_0_ when they are descending, and *A_r_* is their amplitude when they are ascending. Likewise, *D_f_* is the length of the contours of *F*_0_ when they are descending, and *D_r_* is their length when they are ascending. The intensity embodies the height of the audio signal. The intensity of the audio signal is given by:

(4) $$Intensity=10\times log\left(\sum_{n=1}^{N}A^{2}\left(n\right)w\left(n\right)\right)$$

where *w* and *A* are, respectively, the window and the amplitude. The rhythm feature subset includes two main parameters, namely, the raw pairwise variability index (rPVI) and the normalized one (nrPVI), which are convenient for computing the rhythm in a given audio. They expressed as follows:

(5) $$rPVI=\left(\frac{\sum_{k=1}^{M-1}\left|d_{k}-d_{k+1}\right|}{m-1}\right)$$

(6) $$nrPVI=100\times \left(\frac{\sum_{k=1}^{M-1}\left|2\times \frac{d_{k}-d_{k+1}}{d_{k}-d_{k+1}}\right|}{m-1}\right)$$

Furthermore, six other features are computed: standard deviation of the expiration signal, the standard deviation of the expiration signal divided by the mean length, number of expirations in each cry signal, duration of expiration, range of expiration, and average of all expirations in one signal cry signal.

### 2.2. Deep Feedforward Neural Networks

The feedforward neural network (FFNN) [32] is a multilayer perceptron composed of an input layer, a hidden layer, and an output layer. The neurons of end-to-end layers are connected by weights. The signal passes only from the input layer to the first hidden layer, then to the output layer. The output of the network is given by:

(7) $$y_{i}=f\left(\sum_{j=1}^{n}w_{ij}x_{ij}+w_{i0}\right)$$

where *w_ij_* is the weight of the link from neuron *j* of the previous layer to the current neuron *i*, *x_ij_* is the corresponding signal, *w_i_*_0_ is the inherent threshold of neuron *i*, and *f* is an activation (transfer) function. At iteration *i*, the weights of the network are adapted as follows:

(8) $$∆w_{i}=-\gamma_{i}G_{i}$$

where *γ* is the learning rate, and *G* is the gradient. In the deep feedforward neural network (DFFNN), the neurons in the first layer are connected to all the neurons in the next layer, and the data is propagated in the entire network without feedback or a loop process. The major difference between DFFNN and a shallow feedforward neural network (FFNN) is the number of hidden layers used to determine the depth of the network. Specifically, the DFFNN has more than one hidden layer, whilst the shallow FFNN has only one hidden layer.

### 2.3. Optimization Techniques

In machine learning and deep learning, a set of hyperparameters used to configure the neural networks must be set prior to training a model. Indeed, these hyperparameters affect the training process and have a direct influence on the performance of the classification model. In this study, we employ the Bayesian optimization (BO) [33,34] and random search (RS) [35,36] for hyperparameter tuning of the DFFNN.

The Bayesian optimization algorithm is an approximation algorithm effective when the computation task is complex and the number of iterations is large. Specifically, the BO finds the best parameters according to the conditional probability of the performance in the training set using a surrogate, for instance, the estimate of the objective function. Specifically, the classification error is the objective function *f* to minimize. In sum, the BO algorithm essentially involves the following two major phases: (*i*) updating the surrogate model in each iteration to find the best guess and measure the uncertainty of the model and parameters, and (*ii*) finding the next sample point based on the acquisition function used to guide the solution toward the global optimal.

The random search algorithm uses randomness to find the best solution in the area of search. In this regard, randomness is based on the generation of a pseudo-random number based on different probability distributions. The random search algorithm has global convergence capabilities, and it is not significantly affected by the size of the search space or the initial values of the estimates of the parameters. The algorithm used to minimize the objective function *f* is described in the following steps:

(1) Select the starting point *x* and keep it as the current solution.
(2) Produce a random vector *dx* from the parameter space and calculate *f*(*x* + *dx*).
(3) If *f*(*x* + *dx*) < *f*(*x*), save the new solution as the current solution *x* = *x* + *dx*.
(4) Stop if the stopping criterion is achieved. Otherwise, iterate from step 2.

In this study, the learning rate, number of hidden layers, node numbers in each layer, and the activation function are all hyperparameters evaluated by BO and RS algorithms.

## 3. Data and Results

**Figure 2:**
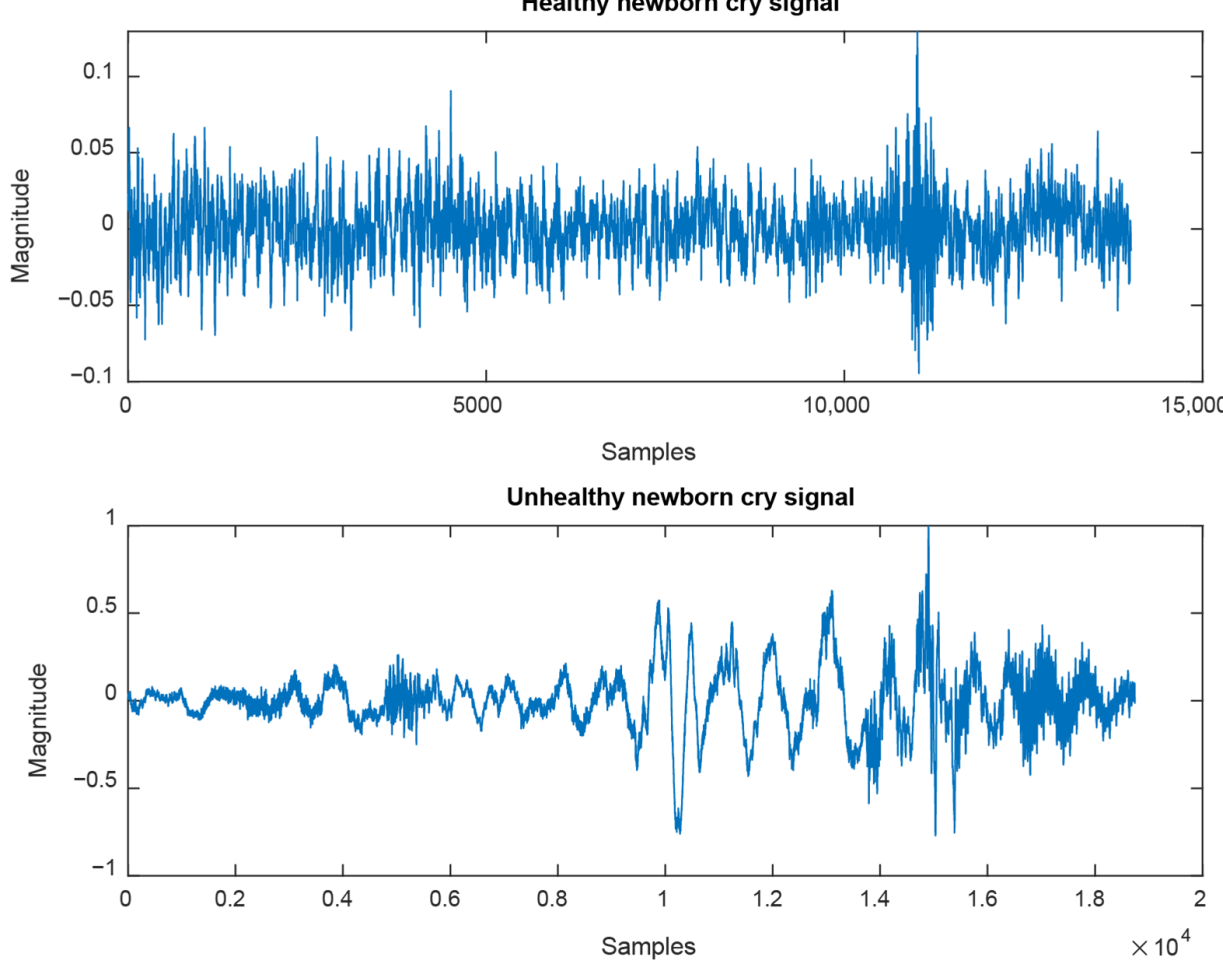
Examples of cry signals are recorded from healthy and unhealthy newborns.

We use a private dataset [30] to analyze and classify cry signals recorded from 763 healthy newborns and 320 unhealthy ones. A two-channel sound recorder is used for all cry signals, with the sampling frequency set to 44.1 kHz, and its length varies between two and three minutes. Figure 2 exhibits examples of healthy and unhealthy cry signals from two different newborns. For experiments, we adopted the ten-fold cross-validation, and we computed the average and standard deviation of the standard performance metrics, namely accuracy (correct classification rate), sensitivity, and specificity. For each model, the objective function to minimize is the classification error.

Although we used 30 iterations for each optimization technique, namely Bayesian optimization and random search, for both BO and RS algorithms, the search space is set as follows: the maximum number of hidden layers is three, the maximum sizes are [219, 256, 287], the activation functions are sigmoid, relu, tanh, and the number of iterations is 30. In this work, we set the search space relatively small to reduce search complexity and processing time.

Table 1 displays the average and standard deviation of correct classification rate (accuracy), sensitivity, and specificity obtained by each DFFNN under a 10-fold cross-validation protocol and with respect to the type of acoustic features and optimization algorithm. As shown, when Bayesian optimization is employed to tune the hyperparameters of the DFFNN, the best accuracy (correct classification rate) is achieved when all acoustic features are used as biomarkers (87.80% ± 0.23), followed by AAM (87.34% ± 0.52), MFCC (78.58% ± 0.17), and prosody (71.72% ± 0.41). This considered, when random search optimization is employed, the DFFNN obtained the highest accuracy when trained with all acoustic features (86.12% ± 0.33), followed by AAM (85.95% ± 0.49), MFCC (80.45% ± 0.20), and prosody (72.92% ± 0.38). Therefore, the combination of all acoustic features helps improve the accuracy to distinguish between healthy and unhealthy newborns under both BO and RS.

In addition, the experimental results indicate that Bayesian optimization yields higher accuracy compared to the random search algorithm when all acoustic features are used for training or only AAM features. Although the random search algorithm outperforms Bayesian optimization when trained with MFFC or Prosody acoustics. In sum, the Bayesian algorithm yields the best results.

Finally, in terms of sensitivity and specificity performance metrics, when Bayesian optimization is adopted to optimize the parameters, the DFFNN classifier achieves 89.17% ± 0.66 sensitivity and 86.12% ± 0.43 specificity when trained with all acoustics, 86.24% ± 0.66 sensitivity and 86.24% ± 0.59 specificity when trained with AAM, 80.71% ± 0.52 sensitivity and 76.11% ± 0.60 specificity when trained with MFCC, and 71.72% ± 0.41 sensitivity and 70.05% ± 0.62 specificity when trained with prosody features. Additionally, when the RS optimization technique is adopted, the DFFNN classifier achieves 89.17% ± 0.66 sensitivity and 86.12% ± 0.43 specificity under all acoustics, 89.01% ± 0.61 sensitivity and 85.98% ± 0.90 specificity under AAM, 79.60% ± 0.50 sensitivity and 75.83% ± 0.77 specificity under MFCC, and 72.85% ± 0.59 sensitivity and 69.55% ± 0.81 specificity under prosody features.

## 4. Discussion

Recently, infant cry signal analysis and classification has received increasing attention where MFCC, Mel Spectrogram, and Tonnetz were used to train residual connections and long short-term memory networks and yielded to an accuracy between 92.92% and 95.98% [39], spectrogram, Mel-scaled spectrogram, and MFCC employed to train neural network with attention mechanism to obtain an accuracy between 87.78% and 93.83% [40], and MFCC and energy features were adopted to train convolutional neural networks to achieve 92.1% [26].

With respect to previous studies [30,31] on the same dataset, the DFFN optimized by Bayesian optimization or by the random search algorithm outperforms all proposed models in [30,31] under all acoustics and under a combination of all of them, as shown in Table 2. For instance, by using AAM features only, the SVM achieved 75.75% accuracy in [30], the SVM tuned by Bayesian optimization achieved 83.62% in [31], and DFFNN obtained 87.34% ± 0.52 and 85.95% ± 0.49 in this study when optimized by BO and RS. Additionally, by using MFCC features only, the SVM achieved 76.50% accuracy in [30], the SVM tuned by Bayesian optimization achieved 74.07% in [31], and DFFNN obtained 78.58% ± 0.17 and 80.45% ± 0.20 in the current work when optimized by BO and RS. When prosody features are used as inputs, the SVM achieved 61.50% accuracy in [30], the SVM tuned by Bayesian optimization achieved 70.65% in [31], and DFFNN obtained 71.72% ± 0.41 and 72.92% ± 0.38 in the current work when optimized by BO and RS. Finally, by using all acoustics as inputs, the SVM achieved 61.50% accuracy in [30], the kNN algorithm tuned by Bayesian optimization achieved 82.88% in [31], and in our study, the DFFNN obtained 87.80% ± 0.23 and 86.12% ± 0.33 under BO and RS.

The presented deep feedforward neural networks, optimized by the BO or the RS algorithm, show effectiveness in distinguishing between healthy and unhealthy newborns based on the analysis and classification of their respective cries. Indeed, these optimized deep learning systems are capable of learning highly complex relationships between the acoustics and the class label. The hidden layers in the DFFNN can learn acoustics and detect the most valuable ones. In summary, both DFFNN-BO and DFFNN-RS in combination with acoustics have demonstrated excellent performance in the classification of newborns compared to most recent works validated on the same database [30,31]. In short, the performance of the DFFNN-BO and DFFNN-RS, as with any machine learning system, depends on the quality of the features, as well as the optimization methods used to configure the systems.

As in the previous works [30,31], our experimental results show that AAM features help improve the accuracy of the classifiers compared to MFCC and prosody features. Therefore, the information about speaking rate, represented by AAM features at different sub-bands, is dominant in improving the accuracy of the classifier to discriminate between the voices of healthy and unhealthy newborns. In short, the experimental results showed that AAM features are effective for the detection of unhealthy cries. In addition, the vocal tract information of the cry signal captured by MFCC features is more important in the classification of newborn cries compared to the prosody used to describe the supra-segmental aspect of cry. Furthermore, the combination of all acoustic features helps to further increase the accuracy of the classifiers. In other words, training the machine learning classifiers with information about speaking rate, vocal tract, and the supra-segmental aspect of cry may help enhance the classification rate in a substantial manner. Finally, in Table 3, we show the processing time of the DFFNN when trained with each category of features and under each optimization technique. As shown, the processing time under BO is significantly below that under RS for each category of features. Therefore, BO is effective in terms of low complexity as it provides a fast-processing time compared to the RS optimization algorithm.

Indeed, Bayesian optimization is used to build a probabilistic model of the objective function based on previous evaluations to update the search directions to find the optimal solution. The random search algorithm is based on random sampling of possible hyperparameter combinations from a determined search space. Then, it evaluates each combination independently, without examining past results. Therefore, the random search algorithm does not guarantee to find the optimal solution and is inefficient as it is used to waste time on unrelated areas of the search space. In sum, the Bayesian optimization is more efficient compared to the random search algorithm, as it reduces the number of trials needed to find optimal hyperparameters and consequently converges to better solutions faster than random search. In other words, BO is known to be effective and fast because it uses probabilities to direct search, and the computation of probability is straightforward.

## 5. Conclusions

In this study, we investigated the effectiveness of acoustic features of newborn cries in improving the accuracy of deep feedforward neural networks to discriminate between healthy and unhealthy subjects. In addition, we compared the performance of the DFFNN under Bayesian optimization and the random search algorithm used for hyperparameter tuning. The experimental results show that the combination of all acoustic features helps achieve the highest accuracy of the DFFNN classifier. In addition, the Bayesian optimization yields higher accuracy compared to the random search algorithm when all acoustic features are used for training or only AAM features. In contrast, the random search algorithm outperforms Bayesian optimization when trained with MFFC or Prosody acoustics. In short, the Bayesian algorithm yields the best results. Furthermore, both the proposed DFFNN-BO and DFFNN-RS outperformed existing models that were tested on the same database. For future work, we seek to consider other deep learning models and feature selection for better improvement of accuracy and understanding of the results.

## Figures and Tables

**Table 1:** Performance measures obtained by DFFNN under BO and RS following 10-fold cross-validation.

	Bayesian Optimization			Random Search		
	Accuracy	Sensitivity	Specificity	Accuracy	Sensitivity	Specificity
All acoustic features	87.80% ± 0.23	89.87% ± 0.51	86.35% ± 0.58	86.12% ± 0.33	89.17% ± 0.66	86.12% ± 0.43
Prosody	71.72% ± 0.41	73.58% ± 0.65	70.05% ± 0.62	72.92% ± 0.38	72.85% ± 0.59	69.55% ± 0.81
MFCC	78.58% ± 0.17	80.71% ± 0.52	76.11% ± 0.60	80.45% ± 0.20	79.60% ± 0.50	75.83% ± 0.77
AAM	87.34% ± 0.52	86.24% ± 0.66	86.24% ± 0.59	85.95% ± 0.49	89.01% ± 0.61	85.98% ± 0.90

**Table 2:** Comparison with previous works on the same database.

Study	Features	Machine Learning	Accuracy
[30]	AAM	PNN	70.70%
[30]	AAM	SVM	75.75%
[31]	AAM	SVM + BO	83.62%
[31]	AAM	kNN + BO	80.07%
Current study	AAM	DFFNN + BO	87.34% ± 0.52
Current study	AAM	DFFNN + RS	85.95% ± 0.49
[30]	MFCC	PNN	68.90%
[30]	MFCC	SVM	76.50%
[31]	MFCC	SVM + BO	72.37%
[31]	MFCC	kNN + BO	74.07%
Current study	MFCC	DFFNN + BO	78.58% ± 0.17
Current study	MFCC	DFFNN + RS	80.45% ± 0.20
[30]	Prosody	PNN	52.10%
[30]	Prosody	SVM	61.50%
[31]	Prosody	SVM + BO	70.65%
[31]	Prosody	kNN + BO	70.43%
Current study	Prosody	DFFNN + BO	71.72% ± 0.41
Current study	Prosody	DFFNN + RS	72.92% ± 0.38
[30]	AAM + MFCC + Prosody	PNN	69.10%
[30]	AAM + MFCC + Prosody	SVM	77.90%
[31]	AAM + MFCC + Prosody	SVM + BO	81.74%
[31]	AAM + MFCC + Prosody	kNN + BO	82.88%
Current study	AAM + MFCC + Prosody	DFFNN + BO	87.80% ± 0.23
Current study	AAM + MFCC + Prosody	DFFNN + RS	86.12% ± 0.33

**Table 3:** Processing time of the DFFNN under each type of features in seconds.

Features	BO	RS
AAM	480.9293	822.0263
MFCC	238.9178	364.2461
Prosody	107.4308	353.5441
AAM + MFCC + Prosody	244.0757	531.0088

## Data Availability

The data source is provided in reference [30].

## References

[1] Al-Nasheri A., Muhammad G., Alsulaiman M., Ali Z., Malki K.H., Mesallam T.A., Ibrahim M.F. (2017). Voice pathology detection and classification using auto-correlation and entropy features in different frequency regions. IEEE Access.

[2] Al-Dhief F.T., Baki M.M., Latiff N.M.A., Malik N.N.N.A., Salim N.S., Albader M.A.A., Mahyuddin N.M., Mohammed M.A. (2021). Voice pathology detection and classification by adopting online sequential extreme learning machine. IEEE Access.

[3] Guo C., Chen F., Chang Y., Yan J. (2022). Applying Random Forest classification to diagnose autism using acoustical voice-quality parameters during lexical tone production. Biomed. Signal Process. Control.

[4] Kim H., Park H.-Y., Park D.G., Im S., Lee S. (2023). Non-invasive way to diagnose dysphagia by training deep learning model with voice spectrograms. Biomed. Signal Process. Control.

[5] Gutiérrez-Serafín B., Andreu-Perez J., Pérez-Espinosa H., Paulmann S., Ding W. (2024). Toward assessment of human voice biomarkers of brain lesions through explainable deep learning. Biomed. Signal Process. Control.

[6] Celik G. (2023). CovidCoughNet: A new method based on convolutional neural networks and deep feature extraction using pitch-shifting data augmentation for COVID-19 detection from cough, breath, and voice signals. Comput. Biol. Med..

[7] Despotovic V., Ismael M., Cornil M., Mc Call R., Fagherazzi G. (2021). Detection of COVID-19 from voice, cough and breathing patterns: Dataset and preliminary results. Comput. Biol. Med..

[8] Bugdol M.D., Bugdol M.N., Lipowicz A.M., Mitas A.W., Bienkowska M.J., Wijata A.M. (2018). Prediction of menarcheal status of girls using voice features. Comput. Biol. Med..

[9] Ye W., Jiang Z., Li Q., Liu Y., Mou Z. (2022). A hybrid model for pathological voice recognition of post-stroke dysarthria by using 1DCNN and double-LSTM networks. Appl. Acoust..

[10] Yao Y., Powell M., White J., Feng J., Fu Q., Zhang P., Schmidt D.C. (2023). A multi-stage transfer learning strategy for diagnosing a class of rare laryngeal movement disorders. Comput. Biol. Med..

[11] Turkmen H.I., Karsligil M.E., Kocak I. (2015). Classification of laryngeal disorders based on shape and vascular defects of vocal folds. Comput. Biol. Med..

[12] Svoboda S., Bořil T., Rusz J., Tykalová T., Horáková D., Guttmann C.R.G., Blagoev K.B., Hatabu H., Valtchinov V.I. (2022). Assessing clinical utility of machine learning and artificial intelligence approaches to analyze speech recordings in multiple sclerosis: A pilot study. Comput. Biol. Med..

[13] Linder R., Albers A.E., Hess M., Pöppl S.J., Schönweiler R. (2008). Artificial neural network-based classification to screen for dysphonia using psychoacoustic scaling of acoustic voice features. J. Voice.

[14] Yagnavajjula M.K., Mittapalle K.R., Alku P., Sreenivasa R.K., Mitra P. (2024). Automatic classification of neurological voice disorders using wavelet scattering features. Speech Commun..

[15] Solana-Lavalle G., Roberto Rosas-Romero R. (2021). Analysis of voice as an assisting tool for detection of Parkinson’s disease and its subsequent clinical interpretation. Biomed. Signal Process. Control.

[16] Lahmiri S., Shmuel A. (2019). Detection of Parkinson’s disease based on voice patterns ranking and optimized support vector machine. Biomed. Signal Process. Control.

[17] Lahmiri S. (2017). Parkinson’s disease detection based on dysphonia measurements. Phys. A.

[18] Hireš M., Gazda M., Drotár P., Pah N.D., Abdul Motin M., Kumar D.K. (2022). Convolutional neural network ensemble for Parkinson’s disease detection from voice recordings. Comput. Biol. Med..

[19] Rosales-Pérez C.A., Reyes-García C.A., Gonzalez J.A., Reyes-Galaviz J.A., Escalante H.E., Orlandi S. (2015). Classifying infant cry patterns by the Genetic Selection of a Fuzzy Model. Biomed. Signal Process. Control.

[20] Chittora A., Patil H.A. (2016). Spectral analysis of infant cries and adult speech. Int. J. Speech Technol..

[21] Sachin M.U., Nagaraj R., Samiksha M., Rao S., Moharir M. (2017). GPU based Deep Learning to Detect Asphyxia in Neonates. Indian J. Sci. Technol..

[22] Lim W.J., Muthusamy H., Vijean V., Yazid H., Nadarajaw T., Yaacob S. (2018). Dual-tree complex wavelet packet transform and feature selection techniques for infant cry classification. J. Telecommun. Electron. Comput. Eng..

[23] Anders F., Hlawitschka M., Fuchs M. (2020). Automatic classification of infant vocalization sequences with convolutional neural networks. Speech Commun..

[24] Ashwini K., Durai Raj P.M., Srinivasan K., Chang C.-Y. (2021). Deep learning assisted neonatal cry classification via support vector machine models. Front. Public Health.

[25] Ting H.-N., Choo Y.-M., Kamar A.A. (2022). Classification of asphyxia infant cry using hybrid speech features and deep learning models. Expert Syst. Appl..

[26] Abbaskhah A., Sedighi H., Marvi H. (2023). Infant cry classification by MFCC feature extraction with MLP and CNN structures. Biomed. Signal Process. Control.

[27] Ozseven T. (2023). Infant cry classification by using different deep neural network models and hand-crafted features. Biomed. Signal Process. Control.

[28] Lahmiri S., Tadj C., Gargour C. (2021). Biomedical diagnosis of infant cry signal based on analysis of cepstrum by deep feedforward artificial neural networks. IEEE Instrum. Meas. Mag..

[29] Lahmiri S., Tadj C., Gargour C., Bekiros S. (2022). Deep learning systems for automatic diagnosis of infant cry signals. Chaos Solitons Fractals.

[30] Matikolaie F.S., Kheddache Y., Tadj C. (2022). Automated newborn cry diagnostic system using machine learning approach. Biomed. Signal Process. Control.

[31] Lahmiri S., Tadj C., Gargour C., Bekiros S. (2023). Optimal tuning of support vector machines and k-NN algorithm by using Bayesian optimization for newborn cry signal diagnosis based on audio signal processing features. Chaos Solitons Fractals.

[32] Goodfellow I., Bengio Y., Courville A. (2016). Deep Learning, Adaptive Computation and Machine Learning Series.

[33] Gelbart M., Snoek J., Adams R.P. (2014). Bayesian Optimization with Unknown Constraints. arXiv.

[34] Garnett R. (2023). Bayesian Optimization.

[35] Rastrigin L.A. (1963). The convergence of the random search method in the extremal control of a many parameter system. Autom. Remote Control.

[36] Bergstra J., Bengio Y. (2012). Random search for hyper-parameter optimization. J. Mach. Learn. Res..

[37] Matikolaie F.S., Chakib T. (2020). On the use of long-term features in a newborn cry diagnostic system. Biomed. Signal Process. Control.

[38] Sarria-Paja M., Falk T.H. (2017). Fusion of auditory inspired amplitude modulation spectrum and cepstral features for whispered and normal speech speaker verification. Comput. Speech Lang..

[39] Qiu Y., Yang X., Yang S., Gong Y., Lv Q., Yang B. (2024). Classification of Infant Cry Based on Hybrid Audio Features and ResLSTM. J. Voice.

[40] Qiao X., Jiao S., Li H., Liu G., Gao X., Li Z. (2024). Infant cry classification using an efficient graph structure and attention-based model. Kuwait J. Sci..

